# Advancements in Mass Spectrometry-Based Targeted Metabolomics and Lipidomics: Implications for Clinical Research

**DOI:** 10.3390/molecules29245934

**Published:** 2024-12-16

**Authors:** Nguyen Ky Anh, Nguyen Quang Thu, Nguyen Tran Nam Tien, Nguyen Phuoc Long, Huy Truong Nguyen

**Affiliations:** 1 Faculty of Pharmacy, Ton Duc Thang University, Ho Chi Minh City 700000, Vietnam; nguyenkyanh@tdtu.edu.vn; 2 Department of Pharmacology and PharmacoGenomics Research Center, Inje University College of Medicine, Busan 47392, Republic of Koreaphuoclong@inje.ac.kr (N.P.L.)

**Keywords:** targeted assay, metabolomics, lipidomics, mass spectrometry, precision medicine

## Abstract

Targeted metabolomics and lipidomics are increasingly utilized in clinical research, providing quantitative and comprehensive assessments of metabolic profiles that underlie physiological and pathological mechanisms. These approaches enable the identification of critical metabolites and metabolic alterations essential for accurate diagnosis and precision treatment. Mass spectrometry, in combination with various separation techniques, offers a highly sensitive and specific platform for implementing targeted metabolomics and lipidomics in clinical settings. Nevertheless, challenges persist in areas such as sample collection, quantification, quality control, and data interpretation. This review summarizes recent advances in targeted metabolomics and lipidomics, emphasizing their applications in clinical research. Advancements, including microsampling, dynamic multiple reaction monitoring, and integration of ion mobility mass spectrometry, are highlighted. Additionally, the review discusses the critical importance of data standardization and harmonization for successful clinical implementation.

## 1. Introduction

Metabolomics represents a field dedicated to the profiling of small endogenous metabolites in biofluids and provides a comprehensive representation of the metabolic phenotype of a biological system, such as cells [1], tissues [2], or organisms [3]. Recent advances in analytical technologies and bioinformatics have significantly increased the utility of metabolomics, facilitating detailed investigations of cellular metabolism [1,4], disease mechanisms [5,6], and biomarker discovery [3,7]. A subset of metabolomics, lipidomics, has garnered considerable attention for its ability to elucidate the roles of lipids in various pathological conditions, including metabolic [8,9] and infectious diseases [10,11,12]. Among the technical platforms utilized in metabolomics and lipidomics, mass spectrometry (MS) stands as one of the most prevalent platforms, especially in clinical science, because it has many advantages. MS can be readily integrated with separation techniques, such as capillary electrophoresis or liquid chromatography (LC), thus reducing the complexity of the sample matrix and the number of compounds in a single detection time and improving compound detection [13]. Additionally, the high sensitivity of MS enables the detection of metabolites at nanomolar or even picomolar concentrations [14]. Furthermore, the flexibility of MS platforms, from low-resolution MS, such as a triple quadrupole (TQ), to high-resolution MS, such as a quadrupole time-of-flight (QTOF), allows their application in different settings.

Generally, metabolomics and lipidomics assays can be categorized into three primary types, targeted, untargeted, and semi-targeted approaches, with targeted and untargeted assays the most widely employed [15]. Untargeted methods aim to capture a broad spectrum of metabolites or lipids within a sample, often offering more extensive metabolite coverage than targeted approaches. However, despite their wide-ranging metabolite profiling capabilities, untargeted assays face notable challenges in clinical and quantitative applications. They often lack quantitative accuracy, display lower reproducibility, and require sophisticated data processing workflows and rigorous quality control. On the other hand, targeted metabolomics and lipidomics have emerged as powerful tools in clinical science, offering significant advantages in terms of quantification, reproducibility, and robustness. Targeted assays often undergo more comprehensive validation than untargeted assays, particularly with respect to critical factors such as calibration linearity, limits of quantification, precision and accuracy, recovery, and sample stability [15]. Thus, targeted methods are crucial for applications of metabolomics and lipidomics in clinical research.

**Figure 1:**
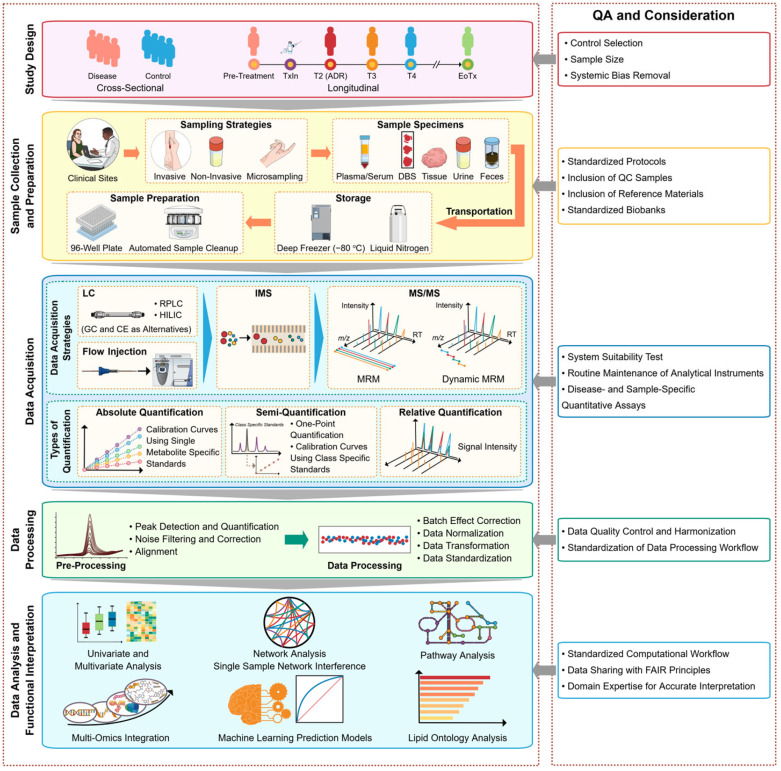
Suggested framework for high-throughput clinical targeted metabolomics and lipidomics studies. The left panel demonstrates the framework, and the right panel includes quality assurances and considerations. Abbreviations: DBS, dried blood spot; LC, liquid chromatography; HILIC, hydrophilic interaction chromatography; RPLC, reverse-phase liquid chromatography; GC, gas chromatography; CE, capillary electrophoresis; IMS, ion mobility spectrometry; MRM, multiple reaction monitoring; FAIR, Findability, Accessibility, Interoperability, and Reusability.

The establishment of a robust and reproducible technical pipeline is vital to ensure the reliability and consistency of data in targeted metabolomics and lipidomics, especially large-scale studies. Advancements in analytical and bioinformatics methods have provided a foundation for reproducible and consistently targeted quantitative metabolomics and lipidomics. In this review, we focused on recent advancements in targeted metabolomics and lipidomics from sample collection to quality control and data analysis for clinical research and implementation. Of note, a suggested framework for targeted metabolomics and lipidomics is depicted in Figure 1.

## 2. Study Design and Typical Applications of Targeted Metabolomics and Lipidomics in Clinical Research

Study design plays a pivotal role in clinically targeted metabolomics and lipidomics. Given that metabolomes are highly dynamic and can be affected by both genetic and environmental factors, no two individuals will have identical metabolomes [16]. These variations necessitate the reduction in confounding factors, such as medications and comorbidities, which may significantly affect metabolites of interest. Thus, key aspects of study design are the clear definition of the biological question and the careful selection of appropriate sample populations and controls. Although it is impossible to control all potential confounders, efforts to minimize their impact are essential. Two primary study designs, cross-sectional and longitudinal, are commonly utilized depending on the research objective. Cross-sectional designs are typically employed to identify biomarkers for diagnosis, whereas longitudinal designs are more appropriate for discovering biomarkers to monitor therapeutic response or treatment outcomes. Targeted metabolomics and lipidomics can be applied for multiple purposes in clinical research. Here, we present some typical applications of targeted metabolomics and lipidomics studies in clinical research.

### 2.1. Identification of Clinical Diagnosis and Risk Biomarkers

Numerous large-scale targeted metabolomics studies have identified potential diagnostic biomarkers. Luo et al. combined untargeted and targeted approaches to analyze serum from 1448 individuals across six centers in China and identified phenylalanyl–tryptophan and glycocholate as promising biomarkers for early hepatocellular carcinoma (HCC) detection [7]. Similarly, Stepien et al. used a commercial kit in a large prospective cohort and reported that metabolites such as lysine, leucine, and glutamine were inversely associated with HCC risk [17]. Vantaku et al. analyzed over 300 metabolites in two bladder cancer cohorts and discovered that the level of metabolites, including taurine and glutamate, varied by ethnicity, suggesting ethnic-specific metabolic signatures [18]. Furthermore, a study in two large prospective cohorts linked disruptions in phosphatidylcholine (PC) and sphingomyelin (SM) metabolism, especially via the arachidonic acid pathway, to increased myocardial infarction risk in healthy adults [19]. Li et al. investigated the associations between 186 targeted metabolites and liver cancer risk in a nested case–control study. Plasma samples used in this study were collected up to 14 years before diagnosis [20]. After confounding factor adjustments, 28 metabolites were linked to liver cancer risk. The key pathways involved included primary bile acid biosynthesis and phenylalanine, tyrosine, and tryptophan biosynthesis. This study suggests a metabolic score as a potential predictor of liver cancer.

Using targeted lipidomics, Chen et al. used LC–MS to quantify 77 sphingolipids in plasma samples from 997 six-year-old children across two cohorts [3]. Their findings indicated that overall elevations in sphingolipids were detrimental; increases in ceramides were specifically associated with asthma risk factors, whereas asthma phenotypes were linked to elevated recycling of sphingolipids. In addition, Eichelmann et al. applied targeted lipidomics in a randomized dietary trial to develop a multilipid score (MLS), reflecting the impact of replacing saturated fats with unsaturated fats in 45 lipid metabolites [9]. The study found that a higher MLS—indicating better dietary fat quality—was associated with a reduction in cardiovascular disease and type 2 diabetes incidence. A simplified version of the MLS, which is called the reduced MLS (rMLS), showed similar associations, with an improved rMLS over 10 years linked to a lower risk of diabetes. In addition, an olive oil-rich diet may be associated with reduced diabetes incidence, particularly in those with an unfavorable reduced MLS preintervention. Overall, these findings suggest the usefulness of targeted metabolomics and lipidomics in biomarker discovery studies.

### 2.2. Assessment of Associated Mechanisms of Metabolic Diseases

The combination of metabolomics and genome-wide association studies has been utilized to investigate the biological mechanisms underlying metabolic diseases. Hartiala et al. integrated genotyping with targeted quantification of betaine to explore genetic variants associated with coronary artery disease and their impact on plasma betaine levels [5]. They identified two loci that were linked to betaine levels, and a variant showed a notable female-specific reduction in the risk of coronary artery disease. In the Framingham Heart Study, genome-wide genotyping and plasma metabolic profiling of 2076 participants revealed that inherited factors had a greater influence on the metabolome than clinical covariates [21]. The study found that 31 genetic loci were linked to plasma metabolites.

Yang et al. developed a high-throughput metabolomics method to investigate metabolomic changes during the menstrual cycle using serum samples from a female cohort [22]. The study revealed that 12.6% of the detected metabolites, including lipids, amino acids, and citric acid, exhibited significant periodic changes correlated with hormonal fluctuations. Additionally, lipidomic methodologies have been applied to study neurodegenerative diseases, such as Alzheimer’s disease and Parkinson’s disease [23]. This highlights the importance of lipids in pathophysiology and the potential for utilizing lipidomics to develop novel strategies for prognosis, early diagnostics, and treatment.

## 3. Sample Collection and Extraction

### 3.1. Sample Collection

Several sample types, including plasma, tissue, or urine, are used in clinical studies. Among them, plasma and serum are currently the most widely used. Both are derived from blood but differ due to their processing. Plasma collection tubes contain additives to prevent coagulation, while serum tubes can include a coagulation enhancer or be entirely free of additives. Moreover, standard plasma still retains platelets to some extent [24]. Serum requires clotting time, typically 30 to 60 min, making plasma easier to handle. Studies have shown metabolite differences, with lipids such as lysophospholipids (LPC) and free fatty acids (FA) generally being more abundant in serum due to enzymatic activity during clotting [24,25,26]. Clotting temperature, duration, enhancers, and different coagulation processes also cause inconsistencies in serum results [24]. Given these inconsistencies, plasma is often preferred over serum in biomarker discovery because of its consistency and ease of handling [24]. Some studies used whole blood instead of plasma or serum. Compared with paired plasma, whole blood presented higher concentrations of most sphingolipids and exhibited lower individual variability, suggesting that whole blood could be an alternative to plasma in biomarker discovery studies [27].

While blood collection has traditionally relied on venipuncture, the use of small amounts of capillary blood offers several advantages over larger venous samples. Capillary blood allows for quicker, simpler sampling and more cost-effective, efficient transport. As a result, recent targeted metabolomics studies have applied blood microsampling for the convenient sampling of plasma, serum, and dried blood spots (DBSs) [28,29]. Common methods of blood microsampling include volumetric dried blood sampling [30,31], dried serum spot sampling [32], and volumetric absorptive microsampling (VAMS) [33], and VAMS is also used for DBS sample collection. DBSs are an emerging sample type in clinical research and are prepared by applying or pipetting whole blood onto a filter card, which is then dried at ambient temperature. Microsampling a DBS is patient friendly and minimally invasive [34]. DBS samples are convenient for storage and transport, as they are stable for many metabolites and carry a low risk of contamination [35,36]. Proper storage with desiccants helps prevent moisture-related degradation [36]. In DBS assays, each DBS spot typically contains approximately 20 µL of blood, although the exact volume per subpunch may vary [37,38,39].

A key challenge in DBSs is the variability due to changes in hematocrits (HCTs), the proportion of red blood cells in the blood. HCTs affect spot size, uniformity, and extraction efficiency, with higher HCTs leading to smaller spots and reduced reproducibility [40,41,42]. To address the HCT-related challenges in DBSs, methods such as precise blood volume combined with whole spot analysis have been proposed [43]. Internal standard (IS) spray has also been suggested as a solution to HCT-based areas and recovery bias [44,45]. Moreover, applying VAMS allows for precise blood volume sampling, potentially minimizing the affection of HCTs to DBS variations [39]. Although VAMS effectively reduces HCT bias while retaining the advantages of DBS, whole blood concentrations have been overestimated in some cases [46]. HCTs also affect analyte recovery, likely because erythrocytes trap analytes, which can be mitigated by optimizing extraction protocols, solvent choice, and sonication [47,48,49]. The method for VAMS extraction, which involved two sonication with a methanol and water mixture, has been reported [48].

The timing of sample collection is also critical in metabolomics studies, as many metabolites are influenced by circadian rhythms and nutritional status. Approximately 15–40% of metabolites, including lipids, fatty acids, and amino acids, fluctuate throughout the day and peak at different times [50,51]. Additionally, fasting or recent food intake can impact metabolite levels, with acylcarnitine and triglyceride levels decreasing post-meal, while amino acid and glucose-related metabolite levels increase. Therefore, consistent timing of sample collection is essential to minimize variations caused by the above factors [52].

### 3.2. Sample Extraction

High-throughput and reproducible sample preparation is essential for clinically targeted metabolomics and lipidomics. Typically, these methods are customized to specific metabolites of interest with common approaches based on organic solvent-based protein precipitation, liquid–liquid extraction, and solid-phase extraction [53]. Robust sample preparation is needed, especially in large-scale metabolomics studies. Notably, for time-sensitive clinical applications, Gehrke et al. presented a fast liquid–liquid extraction protocol for hydrophilic compounds. The protocol can also be universally applied to plasma, whole blood, and red blood cells [54]. With respect to solid-phase extraction, automatic systems employ solid-phase extraction cartridges enabling runtimes of less than 15 s [55]. This method uses automated solid-phase extraction, which is directly linked to a mass spectrometer for quantification. In addition, organic solvent-based extraction, combined with high-throughput 96-well plates, usually seen in commercial kits, and automated 96-well handler technology, is commonly used in large cohorts [22,53,56]. Automated liquid handler technology can provide standardized procedures and consistent sample extraction. While traditional targeted methods focus on a limited set of metabolites, recent advancements have expanded the coverage of analysis. Having a sample preparation technique that allows for broad coverage could benefit studies evaluating complex biological matrices.

In targeted lipidomics, sample extraction can employ either a single solvent or a solvent mixture. The typical methods that use a solvent mixture are the classic chloroform and butanol–methanol method from Löfgren et al. [57]. The butanol–methanol method can be combined with standard 96-well robots, enabling the automatic and efficient extraction of lipids. Compared with traditional chloroform-based extraction methods, the butanol–methanol method for lipid extraction in biofluids and tissue samples has demonstrated superior performance [58,59]. However, if a 96-well plate is unavailable, a two-step liquid–liquid extraction method can be used to increase the robustness of the sample preparation step. A two-step approach, such as the methyl-*tert*-butyl-ether (MTBE) method introduced by Chen et al., could enable simultaneous extraction of both the metabolome and lipidome [60,61]. While single solvent extraction methods are straightforward and easy to perform, those employing solvent mixtures may offer superior coverage [13]. The choice between these methods is often based on the specific objectives of the study. For example, a study focusing on acylcarnitine used a single-step IPA-based extraction [62], whereas investigations of more lipid subclasses involved the use of the MTBE-based technique [61].

### 3.3. Sample Storage

Temperature and pre-centrifugation delays can affect analytical results [63,64,65]. Therefore, laboratories and biobanks must record these conditions systematically. Protocols recommend centrifuging serum for 10 min and plasma for 20 min at 2000× *g* at room temperature (RT) in the processing step. Temperature variations during sample preparation, transport, sorting, and storage can significantly impact the integrity of biological materials [66,67]. In ultralow-temperature freezers (UTFs), temperature differences ranging from 4.7 °C to 20.8 °C across different zones have been observed [65]. To maintain optimal sample conditions, UTFs should be consistently monitored, ideally maintaining a stable −80 °C environment [65,68]. To maximize the usefulness of biospecimens for future analysis, biobank protocols could involve using a CryoPod or equivalent device for transport, sorting samples in a cryogenic workbench, storing them at −80 °C or below in specific UTF temperature zones or below −150 °C in liquid nitrogen vapor, and consistently recording temperature and timestamps throughout the entire process [65].

Although optimal practices for maintaining lipid stability vary depending on the type of specimens, the use of preparation methods that inhibit enzymatic activity and prevent oxidation is generally advised. Additionally, after extraction, lipid extracts should be stored as dried, or in organic solvents containing antioxidants, at −20 °C or lower, in airtight containers shielded from light and oxygen. This approach helps minimize sublimation and prevents molecular changes caused by oxidation, enzymatic activity, and degradation due to light or heat [69]. Low-abundance FAs, diacylglycerols (DGs), and cholesteryl esters (CEs) have been identified as potential markers of degradation. FAs and DGs are produced by lipase activity from triacylglycerols and PCs, while the increase in CEs is likely due to the oxidation of other high-concentration CEs. During sample collection, characterized aliquots could be stored with samples and monitored for DG, FA, and CE species over time. Another biosignature for degradation is the LPC/PC ratio. The concentration ratio of these two lipid subclasses could be used to differentiate between ‘good’ and ‘bad’ quality samples [70]. For sample storage, fasting samples are preferable to non-fasting samples [71].

## 4. Mass Spectrometry-Based Targeted Metabolomics and Lipidomics

### 4.1. Flow Injection

Electrospray ionization (ESI) is often utilized in flow injection-targeted metabolomics and lipidomics [72]. The samples were introduced via an LC autosampler or continuous infusion syringes. The significant advantages of flow injection MS over LC-MS are its shorter runtime, cost effectiveness, and better contamination control, as most contaminants originate from the ion source [13]. Flow injection ESI–MS lipidomic profiling commonly employs a tandem mass spectrometer or high-resolution mass spectrometer, such as a QTOF system. Methods such as selected ion monitoring or multiple reaction monitoring (MRM) are utilized [73]. For the quantification of lipids in clinical samples, flow injection lipidomic often employs class-specific ISs for absolute or semi-quantification, assuming that the mass response is similar among species within the same subclasses. However, certain lipid subclasses, such as CEs, exhibit mass response variation between species, necessitating the use of species-specific response factors [74]. Commercial kits have also employed flow injection lipidomics for high-throughput analysis [75]. Additionally, Schoeny et al. recently introduced a new lipid class quantification strategy using MS with an all-ion fragmentation approach [76]. This strategy measures class-specific fragments across a mass range, achieving low detection limits and making LC-MS widely applicable. Southam et al. presented a workflow for nano-ESI direct-infusion MS in metabolomics and lipidomics that involves extracting metabolites and lipids from tissues and biofluids and then directly infusing the samples into a high-resolution mass spectrometer via a chip-based system [77]. This approach helps minimize ionization suppression or enhancement effects compared with standard ESI.

### 4.2. Reverse-Phase Liquid Chromatography

LC−MS provides several advantages over flow injection MS, such as the ability to separate compounds, and in some cases, isobars and isomers, based on their physicochemical properties, and reduce ion-suppression effects. In targeted metabolomics and lipidomics, reverse-phase LC (RPLC) and hydrophilic interaction chromatography (HILIC) are commonly employed as separation methods. In RPLC-based targeted metabolomics, short microbore columns with fused-core particles and C18-modified sorbents were often used [13]. Common solvents include acetonitrile–water or methanol–water gradients with 0.1% formic acid, and buffer modifiers are often added to optimize selectivity and sensitivity [13]. Other columns are also utilized. For example, a method employing a biphenyl column was developed by Visconti et al. for the absolute quantification of multiple metabolites related to chronic kidney disease [28]. This study used multitargeted internal calibration to address challenges in quantifying endogenous metabolites in complex samples, like serum and plasma. To bypass the lack of a true blank matrix, the response ratio of analytes and stable isotope standards was used for direct quantification. This validated assay was adapted for clinical research.

The mobile phases and additives in targeted lipidomic RPLC methods are optimized for the lipid subclasses of interest. Strong binary mobile phases often use isopropanol or tetrahydrofuran mixed with other solvents [13,78]. Common modifiers include formic acid, acetic acid, ammonium formate, ammonium acetate, or combinations of these salts and acids. For RPLC-targeted lipidomics, C18 or C8 columns are often used. For example, Turtoi et al. used a 150 × 2.1 mm C8 column with a water/acetonitrile mobile phase to quantify polyunsaturated fatty acids and oxylipins in plasma and tissue [78]. Hildebrand et al. highlighted challenges in RPLC-based lipid quantification, particularly the discrepancies between the measurand and ISs in solvent conditions during ionization, which could lead to trueness bias and matrix effects [79]. The study proposes a method to monitor ionization changes during RP gradients.

While RPLC is typically used for separating moderately or less polar metabolites, it can also accommodate highly polar metabolites through ion pair liquid chromatography (IP–LC). IP–LC employs ion-pairing reagents, which feature hydrophobic and charged sections to interact with both the stationary phase and target analytes. Common ion-pairing reagents for cations include hydrochloric acid, perchloric acid, perfluorocarboxylic acids, and alkyl sulfonic acids, while for anions, ammonium and tetraalkylammonium compounds are used. For example, Michopoulos et al. used tributylammonium to profile 130 polar intracellular metabolites, improving the separation of phosphorylated analytes compared to HILIC [80]. In addition, Knee et al. developed an IP–LC–MS method with a C18 column to detect small polar molecules that are difficult to analyze with conventional RPLC using diamylammonium acetate as the ion-pair reagent [81]. Although effective, IP-LC poses challenges, such as ion suppression, decreased sensitivity, and the risk of system contamination, requiring frequent cleaning.

### 4.3. Hydrophilic Interaction Chromatography (HILIC)

Targeted methods using HILIC columns typically employ a gradient, starting with high acetonitrile and gradually increasing water. A small amount of water in the mobile phase is essential for partitioning analytes in the stationary phase. Unlike RPLC, HILIC operates over a wider pH range (2.8–9) and uses modifiers, like ammonium formate or acetate, adjusted with acids or ammonium hydroxide [13]. HILIC is often used for hydrophilic metabolites such as sugars, amino acids, and nucleic acids [82]. Various HILIC stationary phases are available. For example, Tufi et al. investigated five distinct commercial HILIC packing materials for the simultaneous quantification of multiple neurotransmitters [83]. The zwitterionic phase, specifically the ZIC-cHILIC column based on silica gel, demonstrated superior performance, enabling the analysis of 20 analytes within a single 20 min run. Li et al. utilized an amino-based HILIC column to analyze more than 600 plasma metabolites using a high-basicity eluent (pH 9.8) for efficient ionization in ESI mode [84]. This approach not only neutralizes charges from the analyte’s functional groups but also facilitates the transfer of analytes into a single charged form without compromising their ability to ionize in ESI mode. However, the use of such eluents is not feasible with silica-based columns because of the potential destruction of the silica gel. This challenge can be addressed by employing columns with a sorbent based on an organic polymer. Cai et al. presented a targeted metabolomics approach for the quantification of 200 polar metabolites in central carbon metabolic pathways [85]. The workflow also includes ion mode switching based MS detection and is applicable to various biological samples.

The HILIC retention time (RT) for lipids increases with their polarity, leading to narrower peaks within each lipid class. HILIC allows for the coelution of analytes and the respective ISs, ensuring that they ionize under nearly identical conditions. This could reduce the matrix effects and ionization response during LC separation, which is a notable advantage over RPLC [86,87]. However, an isotopic overlap of species is possible because of the coelution of multiple lipid species, and corrections are needed to avoid overestimation [88]. Many targeted lipidomics studies have utilized HILIC as a separation strategy. Munjoma et al. utilized a HILIC platform with a semiquantitative approach to screen 2000 lipids based on over 4000 MRM transitions in human plasma and serum. After the initial screening, absolute quantification was applied to measure over 400 lipids [89]. Of note, the odd chain lipid standards were employed in this study as calibrants. These standards were eluted within the LC peak range of each endogenous lipid class and subjected to the same ionization conditions. This approach enables the use of significantly fewer standards, simplifying the assay and lowering operational costs. Zhang et al. developed a HILIC-based quantitative method with 1200 lipids across 19 subclasses, including both nonpolar and polar lipids [61].

Despite these differences, HILIC and RPLC provided similar results for phosphatidylethanolamine, lysophosphatidylethanolamine, and SM lipids [90]. However, HILIC reported higher quantities of (L)PC lipids than RPLC, particularly for highly unsaturated PC lipids. This overestimation could not be fully explained by lipid loading, IS intensity differences, or isotopic overlap. The varying MS response due to lipid features, such as unsaturation and chain length, suggests that a response factor approach or the use of multiple ISs per lipid class could help. The combination of RPLC and HILIC also allows greater coverage [91]. For example, a tandem MS-based targeted metabolomic was used to quantify more than 200 metabolites related to global metabolism. Both the HILIC and RPLC methods were used in combination with dynamic MRM (dMRM) to increase the coverage and detection of target metabolites [92]. RPLC and HILIC offer different separation mechanisms that can be combined in 2D-LC to better separate metabolites in complex biological samples and distinguish isomeric and isobaric compounds [93]. 2D-LC has found applications frequently in lipidomics studies. Holcapek et al. employed a C18 column for the first dimension to differentiate lipid species by their hydrophobic parts, followed by rapid HILIC separation in the second dimension based on lipid class polarity [94]. In reverse-phase mode, the retention of lipids followed a pattern of equivalent carbon numbers, offering an additional method for identifying lipids. This approach identified 143 lipid species from four categories and ten classes in human plasma.

In addition to HILIC and RPLC, normal-phase LC–MS has also been utilized in targeted metabolomics and lipidomics. Sun et al. developed a high-throughput technique that could allow for the quantification of various oxidized phospholipid species related to ferroptosis in a single injection [95].

### 4.4. Mass Spectrometer

TQ and quadrupole/linear ion trap (QLIT) mass spectrometers are the common instruments for targeted metabolite quantification, typically using selected reaction monitoring (SRM) or MRM [13]. This method is considered the gold standard for absolute quantification because of its wide linear range, high sensitivity, and repeatability. QLIT offers additional MS3 capabilities, providing enhanced structural information. Modern TQ and QLIT instruments feature short dwell times (1–5 ms), supporting hundreds of MRM transitions per LC−MS run and efficient polarity switching. Acquiring at least two MRM transitions per metabolite is recommended for selectivity. This requires an optimized MRM acquisition strategy. By knowing each metabolite’s elution time, MRM transitions are monitored only during specific RT windows, allowing for a greater number of transitions within a single run while maintaining optimal dwell and cycle times [13].

Many metabolomics and lipidomics studies have employed MRM or dMRM. Gertsman et al. developed methods to quantify 55 metabolites in serum via high-resolution MRM [96]. This approach improved accuracy and precision, especially at relatively low concentrations. Gu et al. developed a comprehensive targeted method for serum metabolites using dMRM [97]. This approach allowed for the acquisition of 1890 MRM transitions for 595 metabolites, each with a dwell time of 5 ms. In addition, Yan and Yan presented a notable application in targeted urine metabolomics [98]. They mapped the human urinary metabolome via a directly coupled RPLC-HILIC system, enabling the simultaneous profiling of hydrophilic and hydrophobic metabolites. To construct a comprehensive human urinary metabolome–wide MRM library, both data-dependent and data-independent strategies were employed, resulting in 749 MRM transitions. Other studies have also demonstrated an expanded scope for targeted LC−MS/MS methods, incorporating over 100 metabolites with optimized MRM transitions [99].

Recently, a new approach called parallel reaction monitoring (PRM) has been developed for high-resolution platforms. In this approach, the metabolite precursor is first selected by the first quadrupole and then fragmented using higher-energy collisional dissociation mode. The resulting MS/MS fragments are then scanned in parallel using a high-resolution mass spectrometer. In clinical metabolomics, PRM-based targeted strategies have been successfully developed by Zhou et al., enabling the simultaneous quantification of 237 polar metabolites with excellent quantitative accuracy and reproducibility [100]. A comparison between MRM and PRM revealed that PRM presented higher mass accuracy and provided more tandem mass fragmentation (MS^2^) information than MRM. Wu et al. combined DIA and PRM to investigate the plasma lipids of coronary heart disease patients [101]. Of note, 152 plasma lipids in patients and related conditions were quantitatively analyzed. MRM and PRM methods each have distinct advantages and disadvantages. The advantages of PRM are its higher mass accuracy and specificity, enabled by high-resolution instruments and more information at the MS^2^ level. In contrast, traditional MRM, which is performed on TQ mass spectrometers, lacks full scan precusor data (MS^1^) and MS^2^ data. Of note, MRM methods can also be applied using high-resolution systems. However, PRM has limitations, including slower scan speeds and polarity switching [102]. Overall, PRM and MRM serve as complementary tools for accurate large-scale metabolomic quantification.

Pseudotargeted metabolomics methods have been applied for a combination of advantages from targeted and untargeted assays [103]. Pseudotargeted methods apply full scan mode to acquire ion pairs from pooled biological samples and establish as many MRM pairs as possible for the quantification of metabolites. Finally, the abundances of metabolites in individual samples are determined via the MRM mode of TQ MS. Luo et al. developed a novel strategy for defining MRM ion pairs via a data-dependent strategy under varying collision energy voltages to gather MS^2^ data [104]. Their custom software then selects daughter ions through four steps: precursor ion alignment, MS^2^ spectrum extraction and reduction, characteristic product ion selection, and ion fusion. Wang et al. developed an ion pair selection method using DIA, which demonstrated its ability to provide more comprehensive fragment information, particularly for low-abundance metabolites [105]. Xuan et al. developed a high-coverage pseudotargeted lipidomics method, with a total of 3377 targeted lipid ion pairs and over 7000 defined lipid molecular structures [106]. Pseudotargeted analysis, which is based on pooled biological samples and comprehensive MS^1^ and MS^2^ data, eliminates the need for standards during method development. It retains the precision of targeted methods while significantly expanding metabolome coverage, allowing simultaneous detection of known and unknown metabolites. This makes it a highly promising technique for large-scale sample analysis with acceptable quantitation capability [107]. Compared with fully untargeted methods, these pseudotargeted approaches are particularly valuable for known matrices, as they analyze fewer metabolites but with greater quantitative confidence and reproducibility. However, it is crucial to note that these pseudotargeted MRM methods were not employed as truly quantitative methods, as they do not utilize a series of ISs or standards for all specific metabolites. Consequently, similar to untargeted methods, they were often based on the relative quantification of the measured metabolites.

### 4.5. Ion Mobility Mass Spectrometry

Ion mobility (IM) MS is a powerful technique that allows for the separation of ions according to their characteristics in electric fields, adding an additional analytical parameter to the conventional MS information. Key IM-MS techniques include drift-time, traveling-wave, and field-asymmetric IM spectrometry. IM-MS enables the measurement of collision cross-section (CCS) values, a unique physicochemical property of compounds, enhancing the specificity of targeted assays. Potential applications of IM-MS in targeted assays include separating isobaric compounds and improving selectivity by isolating target metabolites from interferences. Combining the IM with the LC–MS workflow is particularly effective as IM drift times remain consistent across matrices, whereas RTs may be altered by matrix effects. This integration may also reduce the need for extensive sample cleanup. However, ion suppression in the ion source and the limited dynamic range can challenge the accurate quantification of low-abundance metabolites amidst abundant matrices. Some notable studies have combined LC and IM-MS. Lerner et al. utilized a four-dimensional lipidomics approach for high-throughput profiling of various clinical sample types, including plasma, serum, and DBS [108]. In a study by Paglia et al., CCS values were obtained for over 200 lipids in both ESI-positive and ESI-negative modes across different laboratories, demonstrating high reproducibility. The inter-laboratory relative standard deviation (RSD) was less than 3% for 98% of the molecules [109]. Notably, the CCS values were not affected by the MS instrument settings or LC conditions. Baker et al. applied LC-IM-MS for the separation of phospholipid subclasses and reported that combining IMS with existing LC methods could enhance resolution, the signal-to-noise ratio, and specificity [110]. Damen et al. also utilized IM-MS for enhancing lipid isomer separation [111].

Lintonen et al. demonstrated significant advantages in using IMS in combination with flow injection lipidomics [112]. Specifically, the ability of IMS to distinguish isobaric and closely related lipids enables comprehensive lipidomic analysis with accurate identification from low-mass resolution full scan data. Loef et al. evaluated the reproducibility of lipid biomarker measurements in plasma and erythrocytes from 42 individuals over two time points via a commercial kit [113]. Among the 630 lipids analyzed in plasma and 286 in erythrocytes, 78% of the plasma lipids exhibited good to excellent reproducibility, whereas only 37% of the erythrocytes exhibited good reproducibility. Of note, the database for the CSS of lipids includes the METLIN-CCS Lipid Database, providing CCS values for over 750 molecular lipid standards across different lipid classes and ionization modes [114]. The database can provide information for distinguishing between isomeric lipid species, like phosphatidylethanolamines and PC, using CCS values.

## 5. Quality Control

For effective clinical application, it is crucial that targeted metabolomics and lipidomics account for both intra- and inter-laboratory variability [56]. However, achieving standardization and harmonization of methods across different cohorts remains a significant challenge for LC–MS-based techniques [115]. These challenges require the establishment of quality control guidelines and reference databases for both metabolomics and lipidomics [116,117,118,119]. Conducting comprehensive metabolic profiling in large-scale cohorts is vital for establishing a database with reference ranges, which will facilitate comparisons with other laboratories and methods [118,120,121]. Reference databases require the use of widely accepted reference materials, such as the NIST SRM 1950 plasma sample [90,116,122,123,124]. For example, Liu et al. provided reference values of around 200 metabolites using an MS-based method for the harmonization of large-scale metabolomics data [115]. For lipidomics, another reference material is the NIST candidate RM 8231 [125]. Incorporating analysis of reference materials alongside study samples can aid in data normalization and subsequent intra- or inter-laboratory comparisons [126], particularly when dealing with analyte groups that exhibit significant variation in absolute values [118,127].

In targeted metabolomics and lipidomics, quality control (QC) samples are typically either (1) pooled QC samples, made by combining portions of the biological samples, or (2) commercial QC samples with known compositions. QC samples are essential for assessing repeatability and identifying signal drift prior to statistical analysis. The FDA guidelines recommend a relative standard deviation (RSD) of ≤15% for most compounds, or ≤20% near the lower limit of quantification (LLOQ). Additionally, issues such as extract degradation, contamination of the analytical column and ion source, and RT shifts commonly affect instrument sensitivity. To ensure high-quality control, key practices include (1) randomizing sample order, (2) regularly analyzing quality control (QC) samples, (3) using internal standards during extraction or prior to LC–MS analysis, (4) incorporating method blanks to monitor contamination, (5) verifying solvent quality, and (6) checking for carryover by running blank LC–MS injections [13]. For large-scale metabolomics or lipidomics cohort studies, replacing columns and consumables before any quality issues arise is necessary.

Furthermore, ring trials emphasize the importance of system suitability testing, strict protocol adherence, and training of researchers, especially within large cohorts [56,125]. They also highlighted the use of external calibration curves and stable isotope ISs to ensure reproducibility in high-resolution targeted metabolomics and lipidomics. Additionally, incorrect automated integration of analyte peaks can contribute to inter-laboratory variability, underscoring the need for continued software development to improve automated data processing and detect outliers that require manual review [56,128]. Additionally, software solutions, such as MeTaQuaC 0.1.32, employ overview visualization and unsupervised multivariate analysis methods to provide quality control information [128]. Regarding stable isotope ISs, the acquisition of a wide range of isotope standards could be challenging, which can be partially overcome by employing extracts from isotopically labeled organisms, particularly yeast [129,130], for compounds such as amino acids [131].

In targeted lipidomics, the application of appropriate ISs and the utilization of multiple ISs for each lipid subclass are key factors for normalization and reliable quantitative analysis [132]. Since fragmentation in tandem MS modes varies by lipid species, different lipid species may have different response factors, making it necessary to include additional internal standards that represent the diversity within each lipid class [133]. Generally, ^13^C-labeled internal standards are preferred due to their identical retention behavior and the inability of labeled atoms to migrate during fragmentation, unlike deuterium [134]. For automated data normalization via ISs, platforms such as LipidMatchNormalizer have been developed for targeted lipidomics [135]. Another approach for the normalization of lipidomics data is the application of reference materials, which potentially improve the within- and between-batch reproducibility compared to typical ISs normalization [136]. Membrane lipids, such as sphingolipids and glycerophospholipids, are crucial for cellular functions, and changes in their oxidized forms are linked to various diseases. However, commercially available oxidized lipids are scarce, complicating quantitative analysis and biological testing. To address this, researchers use in-house prepared mixtures of oxidized lipids from various sources and oxidant systems [137].

## 6. Data Analysis and Functional Interpretation

### 6.1. Data Processing

Compared to untargeted methods, targeted metabolomics and lipidomics involve simpler data analysis processes and require less extensive training. Typically, mass spectrometer vendors provide data processing software, but specialized tools such as MRMPROBS 3.71, MRM-DIFF 1.0.1, and MRMAnalyzer 3.1.1 are available for targeted MRM methods [85,138,139]. Typically, data processing software from mass spectrometer vendors was used. Another option is Skyline 24.1, a free, open-source tool originally designed for proteomics but now expanded for small molecule analysis [140]. With the rise of IM-MS, new software is needed to process four-dimensional datasets (*m*/*z*, RT, drift time, and signal intensity). Additionally, proper peak alignment is crucial, and tools like DeepRTAlign 1.2.2 (deep learning-based RT alignment tool for large-scale studies) and ReTimeML (machine learning-based automated RT prediction for Cer and SM) offer valuable solutions [141,142].

A challenge in large-scale studies is integrating data from different batches and instruments. Recent advancements have introduced several innovative methods for data correction. Thonusin et al. introduced MetaboDrift 1.1, an Excel-based tool for correcting intensity drift in multi-batch LC–MS datasets [143]. Deng et al. created the WaveICA method, employing wavelet transform and independent component analysis to address batch effects [144]. Additionally, Vaughan et al. developed calibration transfer models to merge data from different LC-MS platforms, facilitating cross-platform data integration [145]. Insight from mass spectrometry-based proteomics could also provide more insights for improving the quality control of quantitative metabolomics and lipidomics [146]. Additionally, novel approaches, such as globally optimized targeted (GOT)–MS, allow broad coverage for metabolic phenotyping via TQ systems [97,147]. GOT-MS and its update can assess various metabolites from many types of biological samples [148].

### 6.2. Data Analysis and Functional Interpretation

Various data mining and statistical methods, including univariate, multivariate analyses, and machine learning models, are applied in targeted metabolomics and lipidomics. Additionally, targeted assays often focus on predetermined specific metabolites associated with particular biological processes. Thus, data interpretation involving pathways and processes relies on domain expertise. Molecular network strategies are also valuable for deciphering the biological patterns underlying phenotypic data. They map and integrate various molecular relationships, facilitating molecule identification and biological interpretation [149]. Here, we provide some notable platforms for the analysis and functional interpretation of targeted metabolomics and lipidomics datasets.

Among the tools for data analysis, MetaboAnalyst 6.0 is one of the most popular platforms that provides comprehensive metabolomics data analysis, including chemometric models, statistical analysis, and machine learning prediction models [150]. The establishment of reference metabolite databases also facilitates data harmonization and biological interpretation. Notable databases including The Human Serum Metabolome Database, which offers data on around 4200 endogenous metabolites in human serum based on analyses from various platforms [151], or The Human Metabolome Database (HMDB) [152], which was established in 2007, include more than 220,000 compound entries—both endogenous and exogenous—along with their molar concentrations, primarily in biofluids, such as blood and urine. The HuMet Repository is a web-based database designed for the assessment of dynamic metabolic responses to six physiological challenges, exercise, 36 h fasting, oral glucose and lipid loads, mixed meals, and cold stress, in healthy individuals [153]. The repository integrates metabolomics data from blood, urine, and breath samples collected from 15 young healthy men at up to 56 time points during these highly standardized tests, which were conducted over four days. The dataset includes 1.1 million data points, covering 2656 metabolites from various biochemical pathways. The platform also allows users to explore metabolites within larger metabolic networks, identify those with similar trajectories, and pinpoint pathways of interest.

Pseudotargeted lipidomics methods allow for the quantification of hundreds of lipid species, making data interpretation complex. Database and lipid ontology analysis platforms have provided solutions for the functional interpretation of complex lipidomics data. Neurolipid Atlas, a lipidomics database from different human iPSC-derived disease models and states, including over 1000 lipid species, facilitates comparative lipidomic research across brain diseases [154]. The database provides a user-friendly knowledge base for the interpretation of lipid dyshomeostasis in neurodegenerative diseases. Lipidomic ontology enrichment platforms can generally be divided into two types, which are platforms that require a database and those that do not [155]. Platforms that do not rely on a database include Lipid Mini-On, LipidSig, and LipidSuite [156,157,158]. Lipid Mini-On and LipidSuite obtain ontology terms using text mining to break down the names of individual lipid species into structural features such as lipid class, saturation level, and carbon chain length. These tools are advantageous because they can analyze lipids that are not yet present in databases, as long as the lipid names adhere to the LIPID MAPS nomenclature. For LipidSig, users can optionally upload a file containing lipid characteristics to obtain ontology terms.

Data sharing is also a crucial aspect of data standardization and harmonization. The Metabolome Annotation Workflow is defined using the Common Workflow Language, which enables it to be executed on various workflow engines [159]. This workflow can act as a guide to applying FAIR practices to bioinformatics or cheminformatics workflows.

## 7. Conclusions and Perspectives

**Figure 2:**
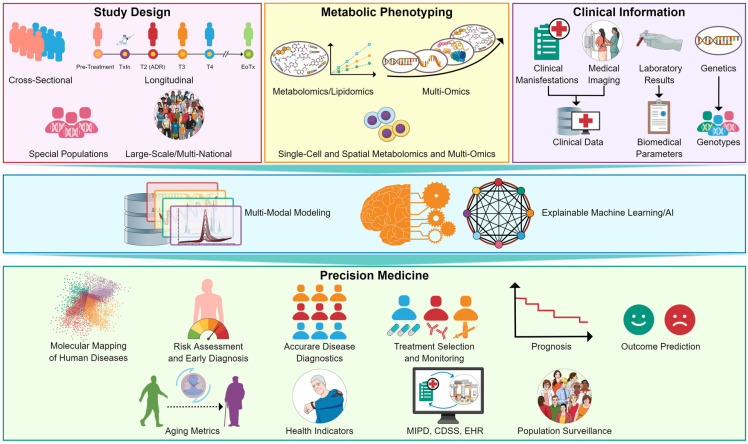
Potential integration of metabolomics and lipidomics in precision medicine. Abbreviations: AI, artificial intelligence; MIPD, model-informed precision dosing; CDSS, clinical decision support system; EHR, electronic health record.

Advances in targeted metabolomics and lipidomics have increased the accuracy and reliability of quantifying endogenous metabolites in clinical samples, paving the way for the integration of metabolite biomarkers in clinical practice. In this review, we present major challenges and recent advances, from sample collection to data analysis and interpretation, to facilitate the implementation of targeted metabolomics and lipidomics in clinical research. This progress also holds significant potential for application in precision medicine. In this context, we illustrate the potential integration of targeted metabolomics and lipidomics with the precision medicine toolbox in Figure 2.

### 7.1. Standardization and Harmonization Efforts Will Provide a Reference Database of Human Metabolite Concentrations, Streamlining Their Application in Clinical Practice

Significant challenges remain before metabolomics and lipidomics can be fully integrated into clinical practice [121,160]. One of the primary factors is the current lack of standardization. Issues such as inconsistent data acquisition, lack of reference concentration ranges of metabolites, and complex data interpretation make it difficult to produce reliable results. Cost-efficient, robust, and reproducible absolute quantitative analysis is needed for clinical applications.

Current practices often adapt regulatory guidelines designed for drug and xenobiotic analysis, which may be overly rigorous for metabolomics applications. Adapting regulatory guidance to create a more practical framework could facilitate the application of targeted metabolomics and lipidomics in clinical settings [161].

While it is recommended to share data alongside publications, only a small fraction of the data from metabolomics and lipidomics studies are currently accessible to the public. In 2016, the FAIR principles for scientific data management and stewardship were introduced to increase the Findability, Accessibility, Interoperability, and Reusability of digital assets [159,162]. Data sharing among studies could facilitate the establishment of reference databases of human metabolite concentrations.

### 7.2. Dried Blood Spots and Microsampling Will Provide a Robust, Cost-Effective, and Convenient Sample Collection Strategy for Clinical Settings

DBS microsampling represents a promising alternative to conventional blood collection methods, offering a minimally invasive, cost-effective solution, especially for specific populations. Its versatility makes it valuable in various clinical studies, including neonatal and pediatric studies, metabolite screening, biomarker research, and sports supervision [163]. This innovation enables safe, accessible diagnostics, making it feasible for both local and global healthcare applications.

### 7.3. Multipurpose Sample Extraction Could Improve Data Integration and Interpretation in Multimodal Omics Studies

Typical approaches to combined proteomic and metabolomic studies often involve preparing samples separately. However, variations from sample preparation processes can cause discrepancies between proteomic and metabolomic data, affecting data integration between these layers. Therefore, consistency between proteomic and metabolomic data can be achieved if both datasets are generated from the same physical sample [164,165]. Processing a single sample for both metabolomic and proteomic analyses can also speed up the sample preparation process and enhance interpretations from the integrated analysis of these modalities [166].

### 7.4. The Application of High-Resolution MS Will Enhance the Accuracy and Specificity of Metabolite Quantification

High-resolution MS presents a powerful tool for enhancing the accuracy and specificity of quantification in targeted metabolomics and lipidomics. Methods employing low-resolution TQ MS often encounter challenges from metabolite interference, where one metabolite can produce a peak in another metabolite’s MRM setting, especially when RTs are similar. Isomeric metabolites are particularly prone to interference. Studies using 334 metabolite standards have shown that about 75% of metabolites can generate signals in the MRM settings of other metabolites, complicating accurate quantification [167]. Additional forms of interference can also arise from the in-source fragmentation of metabolite ions. In targeted lipidomics, isomeric and isobaric overlaps also pose significant challenges for correct quantification and selectivity, especially when low-resolution MS is used [124]. As the field advances, the integration of high-resolution MS into targeted metabolomics and lipidomics workflows is likely to be instrumental in achieving more accurate and reproducible results.

### 7.5. Metabolite Concentrations, Along with Other Data Modalities, Will Enable Early Prediction of Treatment Outcomes via an Explainable and Interpretable AI Model

Timely treatment adjustment significantly benefits patients, but fast and early predictions of on- and off-pharmacological responses are needed. The use of the pharmacometabolomics approach is promising for investigating the relationship of pre-dose metabolite concentrations with Pharmacokinetics/Pharmacodynamics parameters and observable outcomes [168,169]. For example, pre-dose metabolite profiles were used to predict the clearance of busulfan, remoxipride pharmacodynamics, and adverse drug reactions of irinotecan [170,171,172]. A non-linear mixed effects model that combines mechanistic and statistical parts is fundamental [173]. The model might also use data from biosensor-based onsite monitoring for drugs and biomarkers to provide patients with real-time dose adjustments [174,175,176]. In turn, biosensors and multiome profile data can contribute to personal health records, where multiple types of information are already stored (e.g., demographics and medical imaging) [177]. Thus, the contribution of neural network-based models, especially biologically informed and pharmacology informed deep learning models, is necessary. They could use the personal health record data as input nodes in the (dynamical) biology-guided architecture, providing an interpretable model and overcoming their ‘black box’ nature [178,179,180,181]. To potentiate clinical translation, the model also needs an explanation module to examine how much feature change affects the outcome [182]. Finally, developing an AI model in a causal machine learning framework could further predict potential patient outcomes owing to treatments [183]. Altogether, pre-dose metabolite concentrations and other data modalities could be used within an explainable and interpretable AI model for the early prediction of treatment outcomes.

## Data Availability

No new data were created or analyzed in this study. Data sharing is not applicable to this article.
